# Protective immunity against acute toxoplasmosis in BALB/c mice induced by a DNA vaccine encoding *Toxoplasma gondii* elongation factor 1-alpha

**DOI:** 10.1186/s12879-015-1220-5

**Published:** 2015-10-24

**Authors:** Shuai Wang, YuJian Wang, XiaoNi Sun, ZhenChao Zhang, TingQi Liu, Javaid Ali Gadahi, Ibrahim Adam Hassan, LiXin Xu, RuoFeng Yan, XiaoKai Song, XiangRui Li

**Affiliations:** College of Veterinary Medicine, Nanjing Agricultural University, Nanjing, Jiangsu 210095 PR China

**Keywords:** *Toxoplasma gondii*, TgEF-1α, DNA vaccine, Protective immunity

## Abstract

**Background:**

*Toxoplasma gondii* can infect almost all warm-blood animals including human beings. The high incidence and severe damage that can be caused by *T. gondii* infection clearly indicates the need for the development of a vaccine. *T. gondii* elongation factor 1-alpha (TgEF-1α) plays an important role in pathogenesis and host cell invasion for this parasite. The aim of this study was to evaluate the immune protective efficacy of a DNA vaccine encoding TgEF-1α gene against acute *T. gondii* infection in mice.

**Methods:**

A DNA vaccine (pVAX-EF-1α) encoding *T. gondii* EF-1a (TgEF-1α) gene was constructed and its immune response and protective efficacy against lethal challenge in BALB/c mice were evaluated.

**Results:**

Mice inoculated with the pVAX-EF-1α vaccine had a high level of specific anti-*T. gondii* antibodies and produced high levels of IFN-gamma, interleukin (IL)-4, and IL-17. The expression levels of MHC-I and MHC-II molecules as well as the percentages of both CD4^+^ and CD8^+^ T cells in mice vaccinated with pVAX-EF-1α were significantly increased (*p* < 0.05), compared with those in all the mice from control groups (blank control, PBS, and pVAXI). Immunization with pVAX-EF-1α significantly (*p* < 0.05) prolonged mouse survival time to 14.1 ± 1.7 days after challenge infection with the virulent *T. gondii* RH strain, compared with mice in the control groups which died within 8 days.

**Conclusions:**

DNA vaccination with pVAX-EF-1α triggered strong humoral and cellular responses and induced effective protection in mice against acute *T. gondii* infection, indicating that TgEF-1α is a promising vaccine candidate against acute toxoplasmosis.

## Background

*Toxoplasma gondii*, an obligate intracellular protozoan parasite, is responsible for toxoplasmosis in a wide range of hosts including humans, mammals, birds, shellfish and marine mammals [1–5]. In immunocompetent individuals, *T. gondii* infection is usually asymptomatic or solely causes mild symptoms but can result in severe disease, such as ocular toxoplasmosis or encephalitis in immunocompromised patients, and it causes congenital birth defects [6, 7]. In addition to the risk to human health, *T. gondii* infection of agriculturally important animals, such as goats, sheep, and pigs, also causes significant economic losses due to animal abortions and neonatal losses [5, 8].

Currently, chemotherapy is the primary strategy in the treatment of the acute phase of this disease, but it is not effective against *T. gondii* chronic infection [9]. Due to the emergence of drug-resistant parasites and the chemical residues in food that are associated with drug use [10–12], there is an urgent need for an efficient vaccine against toxoplasmosis. During the past decade, anti-*T. gondii* live, attenuated-live, killed and subunit vaccines have been developed [10]. Although the only licensed *T. gondii* vaccine, which is based on the attenuated-live *T. gondii* S48 strain (Toxovax®), can be used to prevent the incidence of abortion in sheep [13], further exploration of its use in other food-producing animals or in humans has been hampered by safety concerns on the possibility of its reversion to a virulence wild type. A DNA vaccine is therefore a better alternative because it does not require the preparation of a whole organism preparation and it has the potential to induce both specific humoral and cellular immune responses as well as long-lasting immunity [10]. In recent years, DNA vaccines against *T. gondii* have been developed and have received considerable attention as good vaccine options [14].

Elongation factor 1-alpha (EF-1α) is highly conserved and ubiquitously expressed in all eukaryotic cells [15–17]. It plays a central role in protein synthesis within eukaryotic cells, and is responsible for aminoacyl-tRNA loading onto the A site of the ribosome [18]. Additionally, it appears to have a number of other functions associated with cell growth, motility, protein turnover, and signal transduction [19]. Recent studies have also suggested that this protein is involved in DNA replication/repair protein networks [20] and apoptosis [21].

In parasites, EF-1α has been implicated in pathogenesis [22] and host cell invasion [23]. *Cryptosporidium parvum* (*C. parvum*) EF-1α protein, which localizes at the apical region of the parasite, mediates cryptosporidial cytoskeletal complex formation. An anti- EF-1α mAb significantly inhibited the host cell invasion by *C. parvum* in vitro. These results indicate that *C. parvum* EF-1α plays an essential role in mediating host cell entry by the parasite and, as such, could be a candidate vaccine antigen against cryptosporidiosis [23]. However, to our knowledge, no studies have evaluated the immunogenicity of *T. gondii* EF-1α (TgEF-1α) and its potential as a vaccine candidate against *T. gondii* infection.

The objective of the present study was to evaluate the potential of TgEF-1α as a vaccine candidate against acute *T. gondii* infection. Therefore, we assessed various immune responses in BALB/c mice that received DNA immunization with a eukaryotic plasmid expressing TgEF-1α.

## Methods

### Ethics statement

The experiments were conducted following the guidelines of the Animal Ethics Committee, Nanjing Agricultural University, China. All experimental protocols were approved by the Science and Technology Agency of Jiangsu Province (approval ID, SYXK (SU) 2010–0005).

### Mice and cell culture

Five-week-old female BALB/c mice were purchased from the Center of Comparative Medicine, Yangzhou University (Yangzhou, China) and maintained under specific-pathogen-free conditions.

Baby hamster kidney (BHK) cells were grown and maintained in Dulbecco’s modified Eagle’s medium (DMEM; Gibco, Beijing, China) supplemented with L-glutamine, 10 % dialyzed fetal bovine serum (FBS; Gibco, USA), 100 IU/ml penicillin, and 100 μg/ml streptomycin in a humidified chamber containing 5 % CO_2_ at 37 °C.

### Parasites and preparation of soluble tachyzoite antigens (STAg)

*T. gondii* RH strain (Type I) was provided by the Laboratory of Veterinary Molecular and Immunological Parasitology, Nanjing Agricultural University, China. The parasites were maintained and collected from the peritoneal cavity of infected BALB/c mice as described previously [24].

Purified tachyzoites were disrupted by three cycles of freezing at −20 °C and thawing at 4 °C. After that, the lysates were sonicated on ice at 60 W/s and centrifuged for 30 min at 12,000 × g. The supernatants were pooled and sterile filtered, and the protein concentration was determined via the Bradford method using bovine serum albumin (BSA) as the standard. STAg was stored in aliquots at −70 °C until use.

### Construction of the DNA vaccine plasmid

The complete open reading frame (ORF) of TgEF-1α (GenBank accession no. XM_002370208.1) was amplified by reverse transcription-polymerase chain reaction (RT-PCR) using designed specific primers (forward primer: 5′- CGC*GGATCC*ATGGGTAAGGAAAAGACTCACATTAAC −3′ and reverse primer: 5′- CCG*CTCGAG*CGAAGCGGTAGATTTGTTCCAAT −3′), in which the *BamHI* and *XhoI* restriction sites, respectively, were introduced and are shown in italics here. Following ligation of the obtained RT-PCR product with the pMD19-T vector (Takara, Dalian, China) to form pMD-EF-1a, the TgEF-1α fragment was cleaved from pMD-EF-1a by *BamHI* and *XhoI* and subcloned into the corresponding sites of pVAXI vector (Invitrogen, Carlsbad, CA, USA). The resulting plasmid was named pVAX-EF-1α. The concentration of the extracted pVAX-EF-1α was determined by spectrophotometry at OD260 and OD280.

### Sequence analysis

The sequence similarity of TgEF-1α to EF-1α from other species was studied using BLASTP and BLASTX (http://blast.ncbi.nlm.nih.gov/Blast.cgi). EF-1α sequences were aligned using MEGA4.0.

### Expression of recombinant plasmids in vitro

Before transfection, BHK cells were transferred to 6-well plates (Corning Costar, Cambridge, MA, USA). When the confluency of the cells reached 80 %–90 %, 5 μg of the recombinant eukaryotic plasmid (pVAX-EF-1α) was used to transfect the cells using Lipofectamine 3000 regent (Invitrogen, Carlsbad, CA, USA) according to the manufacturer’s instructions. The empty vector pVAXI (5 μg) was also transfected into BHK cells as a negative control. Lipofectamine 3000 reagent was respectively mixed with pVAX-EF-1α or pVAXI at a concentration of 10 μg/ml in DMEM without Fetal Bovine Serum (FBS) and antibiotics, and was incubated at room temperature for 30 min. The mixture of lipofectamine and plasmid was then added into BHK cells. The cells were incubated with the transfection mixture for 6 h at 37 °C in the presence of 5 % CO_2_. At the end of this incubation, fresh growing medium was supplemented and plates were returned to the cell incubator for further incubation. After 48 h of incubation, the transfected cells were treated on ice with RIPA lysis buffer (50 mM Tris pH 7.4, 150 mM NaCl, 1 % Triton X-100, 1 % Sodium deoxycholate and 0.1 % SDS) containing 1 mM protease inhibitor phenylmethanesulfonyl fluoride (PMSF) and centrifuged at 13,000 × g for 10 min. The translation of the transfected genes in BHK cells was detected by western blot analysis with anti-*T. gondii* polyclonal antibody (from chicken) as a primary antibody and a horseradish peroxidase (HRP)-labeled goat anti-chicken IgG antibody (SouthernBiotech, Birmingham, AL, USA) as a secondary antibody. Finally, the membrane was soaked in DAB Reagents (Boshide Biotech Co, Wuhan, China) for signal development.

### BALB/c mice immunization and challenge

To assess the immunogenicity of the recombinant plasmids, BALB/c mice were randomly divided into four groups of 30 mice per group. Before vaccination, plasmids were diluted and suspended in sterile phosphate buffered saline (PBS) to a final concentration of 1 μg/μl. All experimental groups were injected intramuscularly (i.m.) three times at weeks 0, 2, and 4 with plasmid DNA (100 μg/each), PBS (100 μl/each) or empty plasmid (100 μg/each), respectively, and one group of mice was not inoculated, which served as a blank control. Blood samples of mice were collected from the tail vein plexus on the day before each vaccination and 2 weeks after the last vaccination. The sera were obtained from the blood samples and stored at −20 °C for evaluation of antibody content and cytokine measurement. At weeks 0, 2, 4, and 6, five mice from each group were sacrificed, and the spleens were collected and used to isolate the splenic lymphocytes for flow cytometry analyses. The remaining ten mice in each group were used for the challenge experiment. Two weeks after the last injection, mice from all four groups were challenged intraperitoneally (i.p) with 1 × 10^4^ tachyzoites of *T. gondii* RH strain. The survival times of the mice were observed and recorded on a daily basis.

### Determination of antibodies by ELISA

The levels of antibodies in mouse sera were determined by enzyme-linked immunosorbent assay (ELISA) as previously described [25]. In brief, the microtiter plates (Corning Costar, Cambridge, MA, USA) were coated overnight at 4 °C with 10 μg /ml STAg in 50 mM carbonate buffer pH 9.6 (100 μl per well). After three washes, the plates were blocked with 3 % Bovine Serum Albumin (BSA) for 2 h at 37 °C and subsequently incubated with the mouse sera diluted 1:10 in PBS for 1 h at 37 °C. HRP-conjugated goat anti-mouse IgA, IgM, IgE, IgG, IgG_1_, or IgG_2a_ (SouthernBiotech, Birmingham, AL, USA) was used as the secondary antibody to detect bound antibodies. Finally, the immune complexes were developed by incubation with 3,3,5,5-tetramethylbenzidine (TMB) for 20 min. The reaction was stopped by adding 2 M H_2_SO_4_, and the absorbance was measured at 450 nm with an automated ELISA reader (Multiskan FC, Thermo scientific, Waltham, MA, USA). All samples were run in triplicate.

### Cytokine assays

To assay cytokine production levels, sera from each experimental group were obtained as described above. Interferon gamma (IFN-γ), interleukin-4 (IL-4), interleukin-17 (IL-17) and transformation growth factor-β1 (TGF-β1) were measured using ELISA kits according to the manufacturer’s instructions (Boster Systems, Wuhan, China). Cytokine concentrations were determined by reference to standard curves constructed with known amounts of mouse recombinant IL-4, IL-17, IFN-γ or TGF-β1. The analysis was performed with the data from three independent experiments.

### Flow cytometry analysis of T cell subsets and MHC molecules

The levels of CD4^+^ and CD8^+^ T cell subsets and the levels of MHC-I and MHC-II molecules in the splenocytes of mice from the four test groups, pVAX-EF-1α, pVAXI, PBS, and blank, were determined using flow cytometry as previously described [26]. Splenocyte suspensions (1 × 10^6^ cells/ml) were dually stained with anti-mouse CD3e-FITC + anti-mouse CD8-PE, anti-mouse CD3e-FITC + anti-mouse CD4-PE, anti-mouse CD3e-FITC + anti-mouse MHC-I-PE or anti-mouse CD3e-FITC + anti-mouse MHC-II-PE (eBioscience, San Diego, CA, USA) for 30 min at 4 °C in the dark. Cell population analyses were conducted with a FACScan flow cytometer with CellQuest software (BD Biosciences, Franklin Lakes, NJ, USA). Lymphocyte specific gating was set according to the forward and side scatter profiles. The percentages of CD4^+^ and CD8^+^ T lymphocytes or, MHC-I and MHC-II molecules in mouse splenocytes were determined as previously described [27].

### Statistical analysis

All statistical analyses were performed by IBM SPSS 20.0 Data Editor (SPSS Inc., Chicago, IL, USA). The differences of the data (e.g., antibody responses and, cytokine production) between all groups were compared by one-way ANOVA. Survival times of the mice were compared using the Kaplan–Meier method. The differences between groups were considered statistically significant if the *p* value was less than 0.05.

## Results

### Successful construction of the eukaryotic expression plasmids

**Fig. 1:**
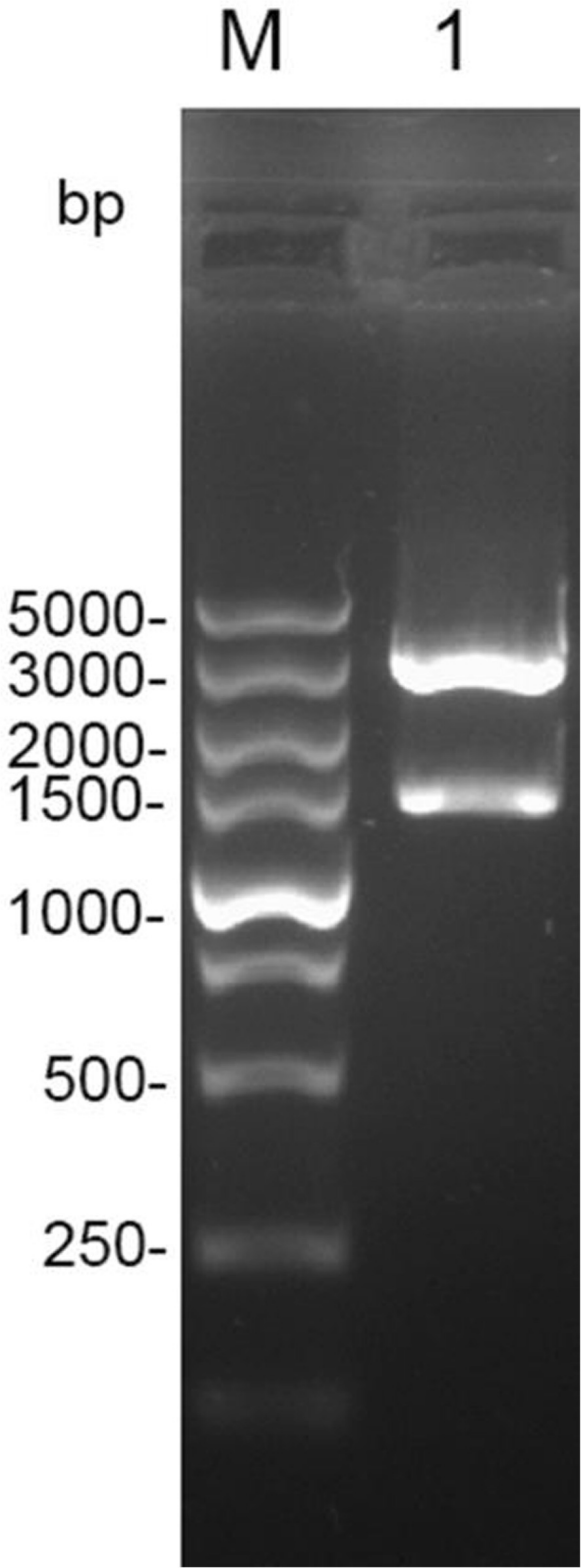
Identification of the recombinant plasmid with restriction enzyme digestion. Lanes 1, the eukaryotic construct pVAX-EF-1α was double digested by BamH I and Xho I enzymes and the product was resolved by 1 % agarose gel to verify a band of size 1347 bp. (M) Represents DNA Molecular marker

The DNA vaccine pVAX-EF-1α was constructed as described in the Methods. To test that the construction was successful, an enzyme digestion was performed with *BamH I* and *Xho I*, yielding a fragment of the expected size, 1,347 pb (Fig. 1). A sequence analysis was also performed and its results showed that the insert in the vector was the ORF of TgEF-1α. Together, these results indicate that the DNA vaccine pVAX-EF-1α was constructed correctly.

### TgEF-1α multiple sequence alignment and cladogram

When compared with the known EF-1α protein sequences on the NCBI database (http://blast.ncbi.nlm.nih.gov/Blast.cgi), the TgEF-1α amino acid sequence had 74 % identity to *Bos taurus* (gi|14422440), *Gallus gallus* (gi|488468), and *Homo sapiens* (gi|15421129), 73 % identity to *Mus musculus* (gi|50797) and *Sus scrofa* (gi|350588388), 70 % to *Capra hircus* (gi|548523658), and 67 % identity to *Canis lupus familiaris* (gi|545523055).

In contrast, the TgEF-1α sequence had 99 % identity to the EF-1α of *T. gondii* ME49 and *T. gondii* GT1, 98 % identity to *Neospora caninum* (gi|401395932), 86 % identity to *Cryptosporidium hominis* (gi|67601420), 85 % identity to *Eimeria acervulina* (gi|557118408), and 83 % identity to *Babesia bovis* (gi|156087152).

**Fig. 2:**
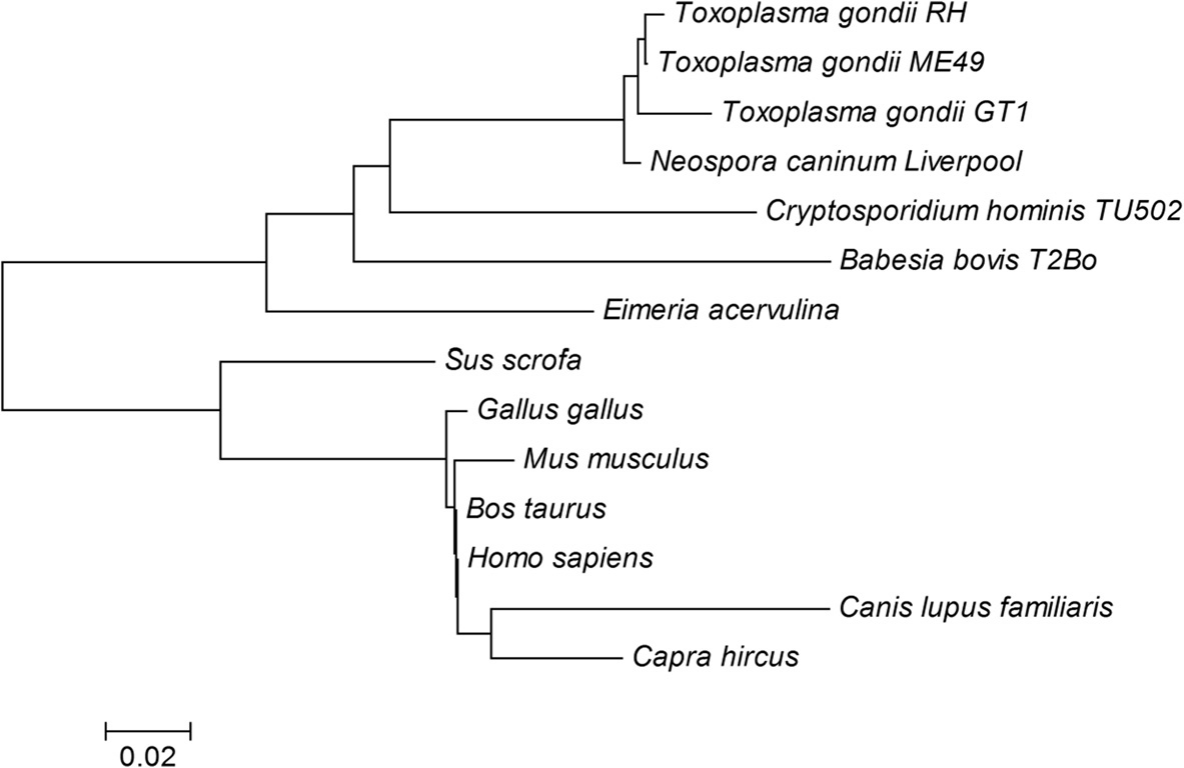
The phylogenetic tree of amino acid sequences between TgEF-1α and those of EF-1α from other species

The phylogenetic tree of amino acid sequences was built using MEGA4.0, and the resulting cladogram (Fig. 2) showed that the kinship of TgEF-1α protein with other species of apicomplexan parasites (*Neospora caninum*, *Babesia bovis*, *Cryptosporidium hominis*, and *Eimeria acervulina*) was high when compared with its kinship with other host species (*Mus musculus*, *Bos taurus*, *Gallus gallus*, *Canis lupus familiaris*, *Capra hircus*, *Sus scrofa*, and *Homo sapiens*).

### Western blot analyses of proteins synthesized in vitro

**Fig. 3:**
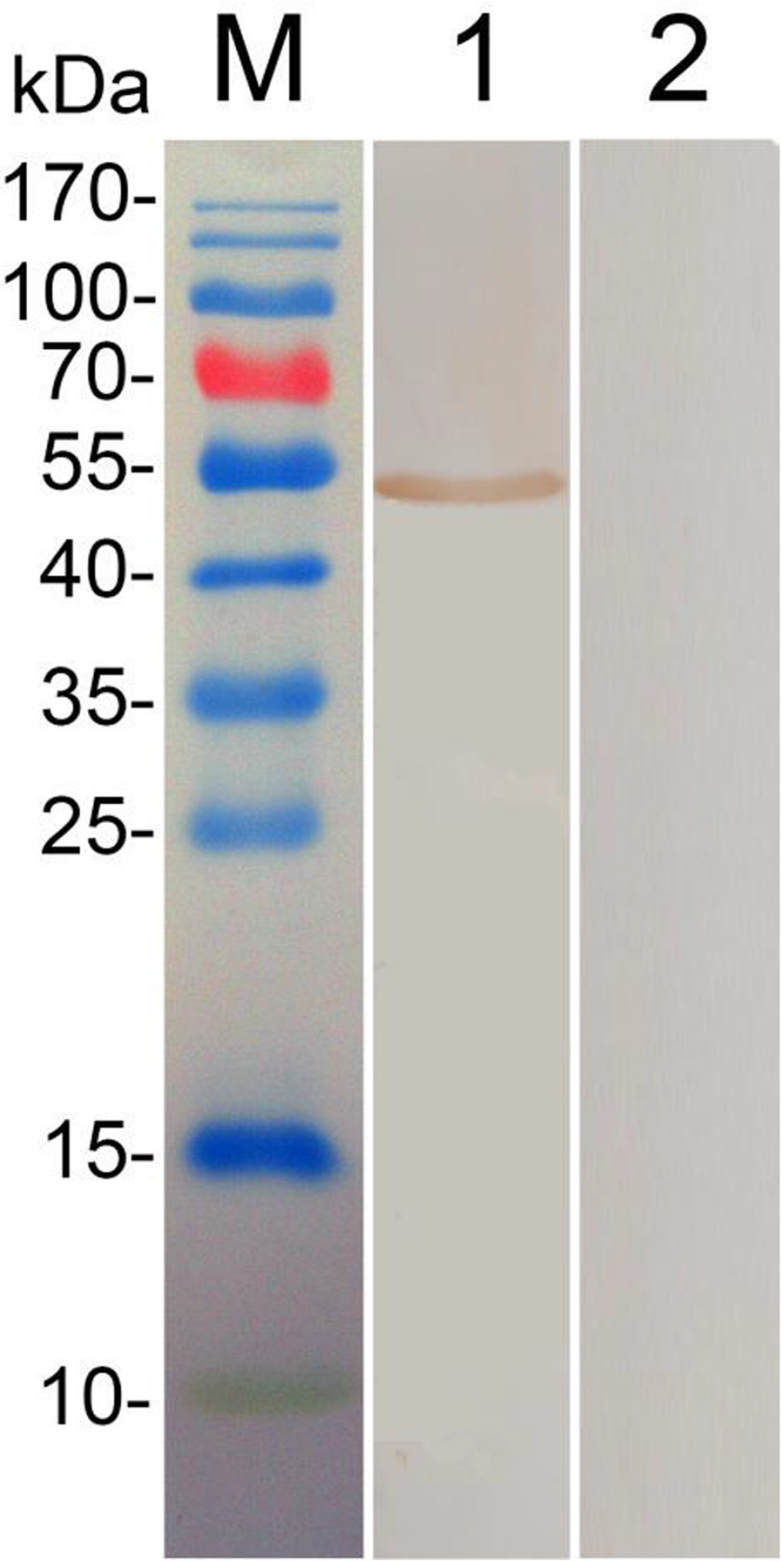
Identification of TgEF-1α in BHK cells by western blot analysis. Lane 1, lysates of BHK cells transfected with pVAX-EF-1α was probed with chicken anti-*T. gondii* sera. Lane 2, Lysates of BHK cells transfected with empty pVAXI vector probed with chicken anti-*T. gondii* sera. (M) Pre-stained protein molecular marker

BHK cells were transfected with pVAX-EF-1α. The lysates of transfected cells were analyzed on immunoblots (Fig. 3). The lysate of BHK cells transfected with pVAX-EF-1α was specifically recognized by serum obtained from a *T. gondii* infected chicken. In contrast, cells transfected with pVAX I were not recognized by this serum.

### Humoral response induced by DNA immunization

**Fig. 4:**
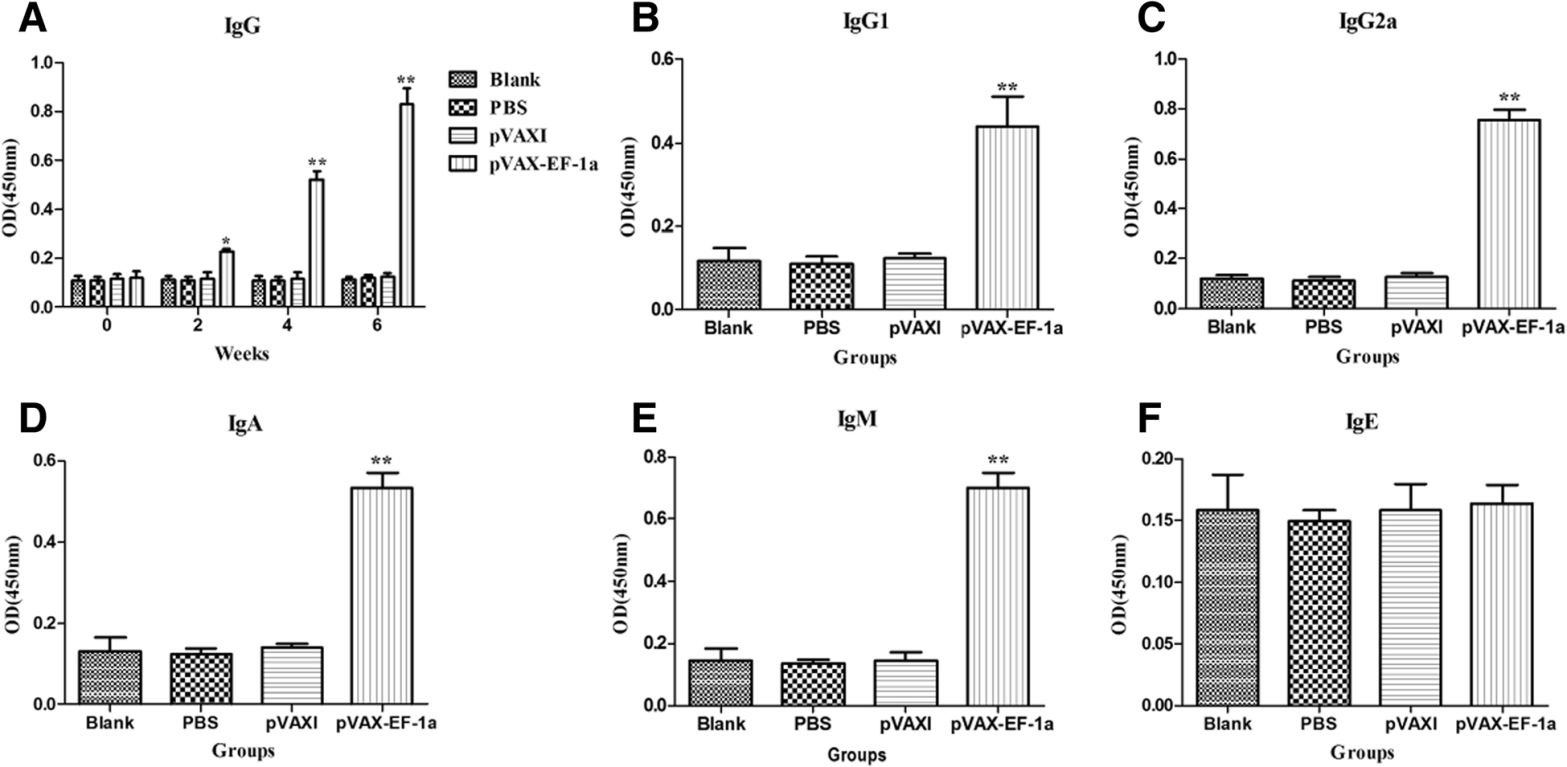
The dynamics of humoral response in BALB/c mice induced by DNA vaccination. **a**. Determination of IgG antibodies in the sera of BALB/c mice immunized with pVAX-EF-1α, pVAXI, PBS and Blank controls on weeks 0, 2, 4, 6. Determination of IgG subclass (**b**) IgG1 and (**c**) IgG2a, (**d**) levels of class IgA, (**e**) levels of class IgM and (**f**) levels of class IgE in the sera of the immunized BALB/c mice two weeks after the last immunization. Results are expressed as mean of the OD450 ± SD. (n = 5) and statistically significant difference (*P* < 0.05) and (*P* < 0.01) are indicated by (*) and (**), respectively

To evaluate the level of antibody induced by three consecutive DNA immunizations, we collected serum samples prior to each vaccination as well as at 2 weeks after the last immunization. Then, we performed ELISAs to determine the total IgG, the distribution of IgG_1_ and IgG_2a_ isotypes, and the IgA, IgM, and IgE two weeks after the last immunization. Compared with the three control groups, a significantly higher level of IgG antibody was detected in the sera of mice immunized with pVAX-EF-1α (*p* < 0.01), and the OD values of IgG continuously increased with successive DNA immunizations. There were no statistically significantly differences in the IgG levels among the three control groups (Fig. 4a).

The levels of IgG_1_ and IgG_2a_ in the pVAX-EF-1α group were also the highest in comparison with those in the three control groups (*p* < 0.01) (Fig. 4b & c). An apparent predominance of IgG2a over IgG1 was observed in immunized mice, demonstrating that a Th1-type cell immune response was elicited by immunization with pVAX- EF-1α.

Regarding the IgA, IgM, and IgE levels, in comparison with the levels in the control groups, the dynamics of IgA and IgM demonstrated significantly higher OD values (*p* < 0.01) in the immunized group: 0.533 ± 0.037 and 0.701 ± 0.048, respectively (Fig. 4d & e). However, IgE activity showed no significant differences between any of the groups at the time of evaluation (Fig. 4f).

### Cytokine production

**Fig. 5:**
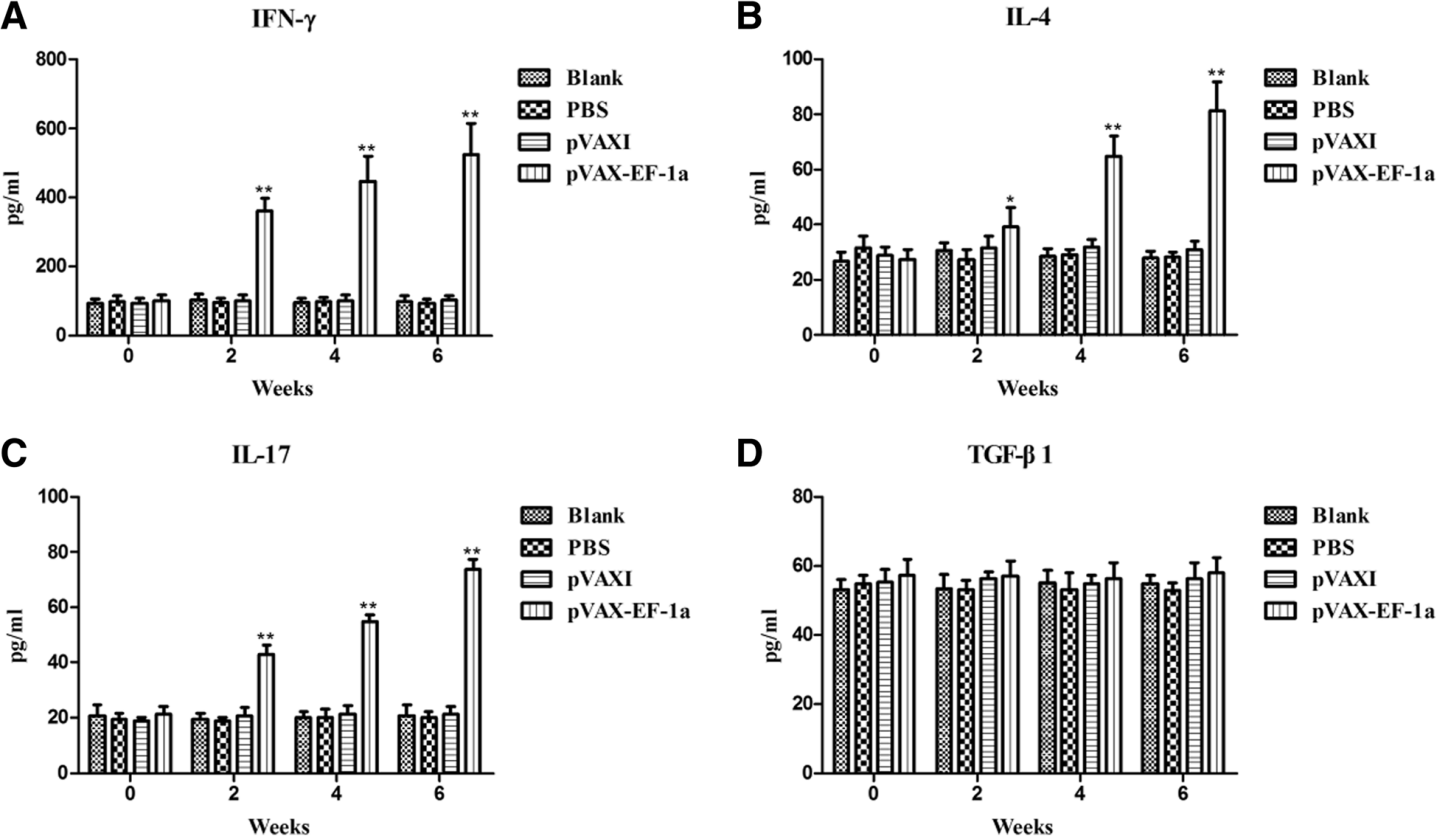
Cytokine production. Antibody-captured ELISA was used to determine the production levels of (**a**) IFN-γ, (**b**) IL-4, (**c**) IL-17 and (**d**) TGF-β1, in sera samples (n = 5) collected at weeks 0, 2, 4 and 6, and the comparison results were expressed as means ± SD of pg/ml. The asterisk designates statistically significant differences (* represents *p* < 0.05; ** represents *p* < 0.01) between groups. Results presented here were from three independent experiments

Sera samples collected at weeks 0, 2, 4, and 6 were used to measure the amounts of IFN-γ, IL-4, IL-17, and TGF-β1 produced in the different experimental groups. As shown in Fig. 5a, significantly higher levels of IFN-γ were observed in mice immunized with pVAX-EF-1α compared to the three control groups at 2, 4, and 6 weeks post-immunization. Small amounts of IL-4 and IL-17 were also secreted by mice in the pVAX-EF-1α group post-immunization, which although modest, were still significantly higher than the amounts secreted by mice in any of the three control groups post-immunization (*p* < 0.05) (Fig. 5b & c). In contrast, the levels of TGF-β1 displayed no significant changes between any of the groups at similar times of evaluation (Fig. 5d).

### T lymphocytes CD4^+^ and CD8^+^subpopulations and MHC molecule changes

**Fig. 6:**
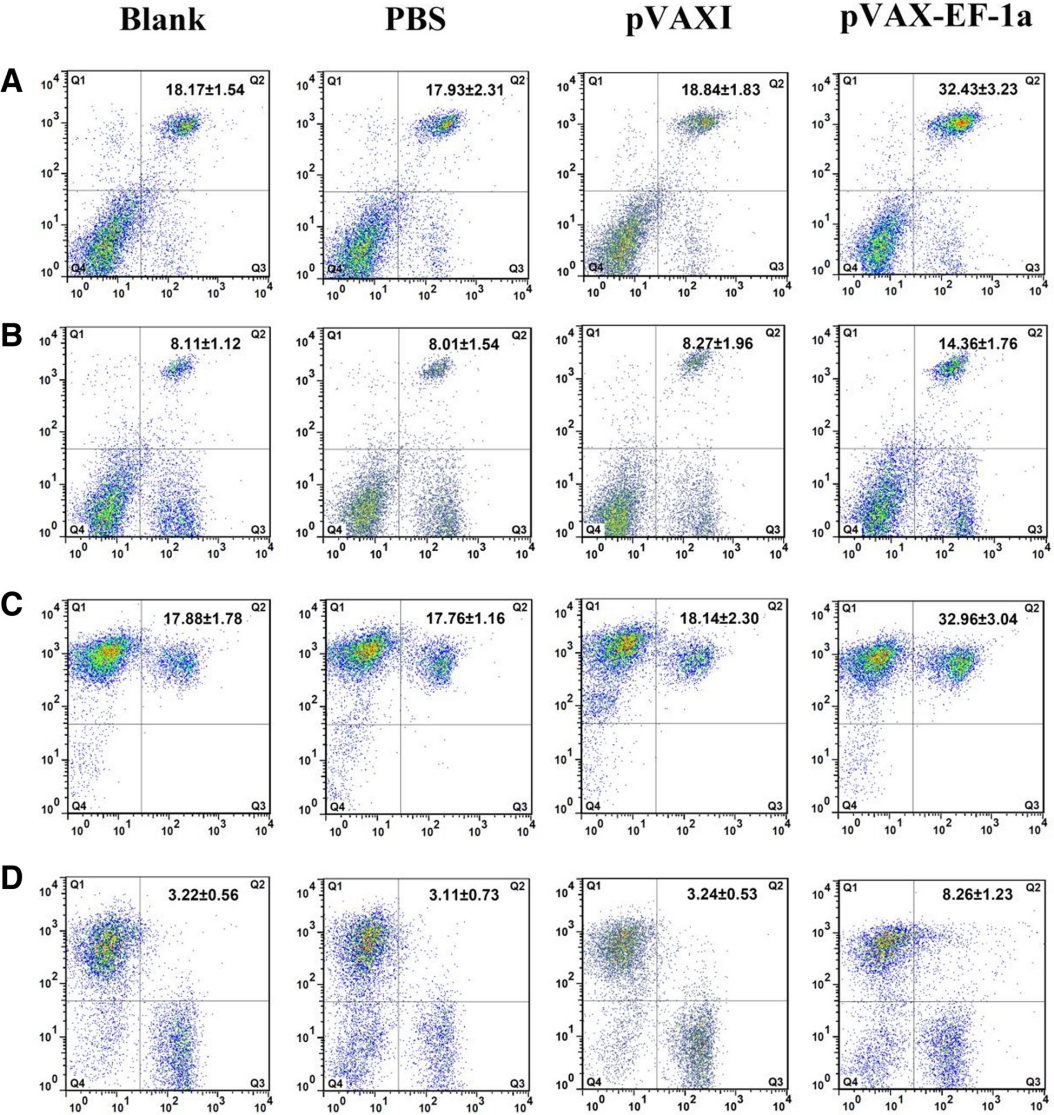
Flow cytometry strategy. Detection of T lymphocyte subpopulation and MHC molecules using flow cytometry technique (CD3 gated), **a** CD4^+^ T lymphocytes (CD3^+^CD4^+^, region Q2). **b** CD8^+^ T lymphocytes (CD3^+^CD8^+^, region Q2). **c** MHC-I molecules (CD3^+^MHC-I, region Q2). **d**. MHC-II molecules (CD3^+^MHC-II, region Q2)

As illustrated in Table 1 and Fig. 6a, the percentage of CD4^+^ T cells was significantly increased (*p* < 0.01) in the pVAX-EF-1α group 2 weeks after the last vaccination (32.43 ± 3.23), compared with that in the pVAXI (18.84 ± 1.83), PBS (17.93 ± 2.31) and the blank (18.17 ± 1.54) groups. Meanwhile, pVAX-EF-1α group showed the highest percentage of CD8^+^ T cells after the last immunization (14.36 ± 1.76), which was significantly different at (*p* < 0.01) when compared with the percentages in the control groups: pVAXI (8.27 ± 1.96), PBS (8.01 ± 1.54) and blank (8.11 ± 1.12) (Table 1 and Fig. 6b).

**Table 1:** Flow cytometry analysis of the percentages of T lymphocyte subsets

Marker (%)	Time point	Groups (n = 5)			
		Blank	PBS	pVAXI	pVAX-EF-1a
CD4^+^	Week 0	18.01 ± 2.95	18.53 ± 2.76	18.23 ± 2.85	17.98 ± 2.45
	Week 2	18.41 ± 1.85	18.55 ± 1.96	17.90 ± 2.53	28.18 ± 3.05^a^
	Week 4	18.03 ± 2.84	18.80 ± 2.88	18.15 ± 1.93	30.46 ± 3.59^a^
	Week 6	18.17 ± 1.54	17.93 ± 2.31	18.84 ± 1.83	32.43 ± 3.23^a^
CD8^+^	Week 0	8.20 ± 1.55	8.13 ± 1.28	8.47 ± 1.81	8.32 ± 1.65
	Week 2	8.32 ± 1.94	8.00 ± 1.48	7.94 ± 1.15	13.23 ± 2.41^a^
	Week 4	8.37 ± 2.07	8.13 ± 1.58	8.25 ± 1.52	12.50 ± 3.11^a^
	Week 6	8.11 ± 1.12	8.01 ± 1.54	8.27 ± 1.96	14.36 ± 1.76^a^

Data are presented as the mean ± SD (n = 5). ^a^ represents statistically highly significant difference (*p* < 0.01) as compared with control groups: Blank, PBS and pVAXI

After the last immunization, the immunized group had a significantly higher percentage of MHC-I^+^ cells (32.96 ± 3.04), in contrast to pVAXI (18.14 ± 2.30), PBS (17.76 ± 1.16), and blank (17.88 ± 1.78) groups (Fig. 6c). The amount of MHC-II^+^ cells increased over time in the vaccinated group (Fig. 6d), starting at week 2 of the experiment and reaching a peak point (8.26 ± 1.23) at week 6 of the experiment that was significantly higher (*p* < 0.01) than the levels of MHC-II^+^ cells in the control groups: pVAXI (3.24 ± 0.53), PBS (3.11 ± 0.73), and blank (3.22 ± 0.56) (Table 2).

**Table 2:** Dynamics of MHC-I and MHC-II molecules in spleen lymphocytes

Marker (%)	Time point	Groups (n = 5)			
		Blank	PBS	PVAXI	PVAX-EF-1a
MHC-I	Week 0	18.19 ± 2.46	17.17 ± 2.62	16.99 ± 1.38	17.29 ± 2.34
	Week 2	16.55 ± 2.15	17.13 ± 1.91	17.06 ± 2.44	24.23 ± 3.33^a^
	Week 4	17.64 ± 1.82	18.02 ± 1.91	17.62 ± 1.46	31.44 ± 3.54^a^
	Week 6	17.88 ± 1.78	17.76 ± 1.16	18.14 ± 2.30	32.96 ± 3.04^a^
MHC-II	Week 0	3.07 ± 0.97	2.88 ± 0.37	3.31 ± 0.49	3.27 ± 0.61
	Week 2	2.99 ± 0.71	3.17 ± 0.38	3.07 ± 0.97	4.84 ± 1.86^a^
	Week 4	3.25 ± 0.45	3.17 ± 0.38	3.27 ± 0.59	7.52 ± 1.42^a^
	Week 6	3.22 ± 0.56	3.11 ± 0.73	3.24 ± 0.53	8.26 ± 1.23^a^

Data are presented as the mean ± SD (n = 5). ^a^ represents statistically highly significant difference (*p* < 0.01) as compared with control groups: Blank, PBS and pVAXI

### Assessment of the protective efficacy of DNA immunization of mice against acute *T. gondii* infection

**Fig. 7:**
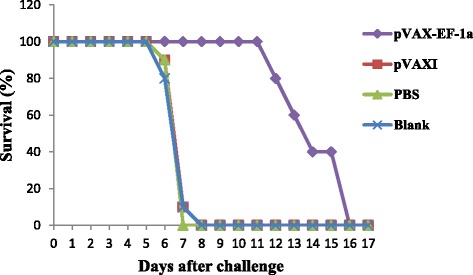
Survival curve of mice after challenge infection with *Toxoplasma gondii* RH strain. Mice were challenged with 10^4^ tachyozoites of the RH strain intraperitoneally two weeks after the third immunization

To analyze the protective efficacy of DNA vaccination against *T. gondii*, we assessed the survival time of mice after infection with 10^4^ tachyzoites of the virulent RH strain. The mice immunized with pVAX-EF-1α had a significantly prolonged survival time (14.1 ± 1.7 days, *p* < 0.05) compared with the control mice that received either pVAX I or PBS. All of the mice in the control groups died within 8 days (Fig. 7).

## Discussion

In the present study, a DNA vaccine encoding TgEF-1α was constructed and its capacity to induce highly significant immune responses and subsequent protection of BALB/c mice against a lethal dose challenge of the highly virulent *T. gondii* RH strain was demonstrated.

Humoral immunity in the form of antigen specific IgG antibodies seems to be important in controlling *T. gondii* invasion [28]. These specific antibodies can inhibit parasite attachment to the host cell receptors and can promote macrophages to kill intracellular parasites, which seem to be important in controlling *T. gondii* infection and its reactivation [29]. Here, the high level of anti-*T. gondii* IgG antibody was induced in the experimental group (pVAX-EF-1α vaccinated mice) compared with the levels in the three control groups (*p* < 0.05). Further analyses of the IgG subclasses (IgG_1_ and IgG_2a_) revealed a predominance of IgG_2a_ over IgG_1_, indicating that pVAX-EF-1α could elicit a Th1-biased humoral immune response, which is considered to play a critical role in the protective immunity against *T. gondii* [30, 31]. This result is consistent with those described in previous studies [31–33].

Immunoglobulins IgA, IgM, and IgE have also been reported to participate in the immunological responses against *T. gondii* infection [34–37]. However, less attention has been paid to these immunoglobulins during vaccination trials, where the focus has been almost entirely directed to IgG [25, 38, 39]. IgA is important in mucosal immunity to oral infection with *toxoplasma* cysts [40]. Since antibodies of this isotype are important in preventing re-infection with *T. gondii*, inducing IgA may be a major strategic aspect of vaccine development [35]. IgM has been reported to increase the phagocytic capacity of neutrophils and to be potentially capable of activating the complement cascade, which may result in killing of the parasite [41, 42]. Moreover, it could reduce the spread of the parasite by blocking cell invasion [43]. In our study, high titers of IgA and IgM were detected in the immunized group, indicating that TgEF-1α had successfully induced the release of these antibodies as part of the response generated after immunization.

The induction of IgE has been previously recognized during the *T. gondii* infection [37, 44]. However, our data revealed no significant traces of this immunoglobulin after vaccination with TgEF-1α, an observation consistent with findings from previous studies [25, 45, 46].

Cytokines play an important role in the activities of T helper (Th) cells. It is well known that IFN-γ is the central cytokine that is responsible for resistance against *T. gondii* during both the early and late stages of infection [47–50]. Compared with the three control treatments, immunization with pVAX-EF-1α enhanced the Th1 mediated immunity by inducing a high level of IFN-γ. In addition, as a factor of Th2 type immune response, a slight increase of cytokine IL-4 was also observed. These results therefore demonstrated that pVAX-EF-1α could elicit strong Th1 cellular immune responses, which is essential for cell-mediated immunity and resistance against intracellular pathogens [51, 52]. Similarly, several other studies reported a distinct pattern of Th1 response to DNA-based vaccines against *T. gondii* [30, 45, 53].

IL-17 is produced by Th17 cells and functions as a tissue inflammatory modulator [54]. This cytokine is also involved in the resistance against *T. gondii* infection [55, 56]. In this investigation, a significant increase of IL-17 concentration was detected in pVAX-EF-1α vaccinated mice over the levels in the control group mice. This finding indicated that TgEF-1a was capable of inducing Th17 differentiation and resulted in an inflammatory reaction. Additional research regarding this T helper type of cells is required to further clarify the roles played by this cell type and the cytokines it releases during vaccination against *T. gondii*.

The cytokine TGF-β produced by regulatory T cells (Treg cells) plays an integral role in regulating immune responses. TGF-β has pleiotropic effects on adaptive immunity, especially in the regulation of effector and regulatory CD4^+^ T cell responses [57]. TGF-β signaling was activated in astrocytes during toxoplasmic encephalitis and inhibition of astrocytic TGF-β signaling increases immune cell infiltration, uncouples proinflammatory cytokine and chemokine production from the central nervous system parasite burden, and increases neuronal injury [58]. Our results showed that no significant production of TGF-β1 was detected in pVAX-EF-1α vaccinated group. This finding could be due to its antagonistic relationship with other cytokines, such as IFN-γ [59–61].

Among the subclasses of T lymphocytes, CD4^+^ and CD8^+^ T lymphocytes play an important role in host resistance to *T. gondii* infection [62]. CD3^+^ CD4^+^ CD8^−^ is the surface marker of T helper (Th) cells that can participate in the adaptive immune responses, while CD3^+^ CD8^+^ CD4^−^ is expressed on cytotoxic T cells (CTLs) which is classified as a pre-defined cytotoxic role player within the immune system [63]. In the present study, we observed differences in the relative proportions of CD4^+^ and CD8^+^ T cells between the vaccinated and control groups. Specifically, we found an increase in both of these components in immunized mice. This observation suggests that immunization with pVAX-EF-1α may induce the activation of both CD4^+^ and CD8^+^ T cells, which may contribute synergistically to cytotoxic activity against *T. gondii* infection.

DNA vaccines stimulate both the exogenous (MHC class II restricted) and the endogenous (MHC class I restricted) antigen presentation pathways [53]. This study found that both the MHC class I and MHC class II molecules were up-regulated in the pVAX-EF-1α vaccinated group compared with their levels in the control groups. The enhancement of MHC class I expression might be related to the significant increase of IFN-γ that we observed in the pVAX-EF-1α vaccinated group. IFN-γ up-regulates MHC class I expression as well as antigen processing and presentation on cells via activation of Janus kinase /Signal transducer and activator of transcription 1 (JAK/STAT1) signal transduction pathway. Briefly, MHC class I antigen presentation-associated gene expression is initiated by interferon regulatory factor-1 (IRF-1). IRF-1 expression is initiated by phosphorylated STAT1. IFN-γ binds to IFN receptors, and then activates JAK1/JAK2/STAT1 signal transduction via phosphorylation of JAK and STAT1 in cells [64, 65]. An up-regulation of MHC class I molecules would generally facilitate the CD8^+^ cytotoxic T cell killing of *T. gondii*-infected cells and may serve to limit parasite multiplication [66]. The up-regulated MHC-II molecules would be able to present more *T. gondii*-derived antigenic peptides to CD4^+^ T cells and induce stronger immune responses during *T. gondii* infection, leading to inhibition of the parasite [62].

To evaluate the protection efficacy of the DNA vaccine, immunized BALB/c mice were intraperitoneally challenged with 1 × 10^4^ tachyzoites of the highly virulent *T. gondii* RH strain. In this acute infection model, mice immunized with pVAX-EF-1α had a significantly prolonged survival time, demonstrating the protective efficacy of this vaccine, which was similar to the results of previous studies employing several other single gene DNA vaccines [25, 45, 67]. but it is not as good as that of other pVAX vaccination candidates such as pVAX-eIF4A [68], pVAX-ROP9 [33] and pVAX-ROP8 [69].

## Conclusion

In summary, this study revealed that the DNA vaccine pVAX-EF-1α encoding TgEF-1α can trigger strong humoral and cellular immune responses, and can induce a prolonged survival time against lethal *T. gondii* challenge. Although TgEF-1α elicited only partial protection against acute toxoplasmosis, it could be used as a potential vaccine candidate in further studies of multi-component *T. gondii* vaccines against toxoplasmosis.
